# Meta-analysis of the effects of different exercise modes on cardiac function and peak oxygen uptake in patients with type 2 diabetes mellitus

**DOI:** 10.3389/fphys.2024.1448385

**Published:** 2024-11-12

**Authors:** He Jianghua, Ma Feier, Zhu Dong, Li Qiuying, Wen Ya, Wang Yan

**Affiliations:** ^1^ School of Sports Medicine and Rehabilitation, Beijing Sport University, Beijing, China; ^2^ College of Sports Medicine and Healthcare, Hunan University of Medicine, Huaihua, China

**Keywords:** type 2 diabetes mellitus, exercise, cardiac function, peak oxygen uptake, systolic function, diastolic function

## Abstract

**Background:**

The benefits of exercise for primary and secondary prevention of cardiovascular events have been reported in patients with type 2 diabetes mellitus (T2DM). However, the effects of exercise on cardiac structure and function require clarification.

**Methods:**

A literature search for clinical studies reporting on the effects of exercise on cardiac structure, cardiac function, and VO_2_peak in T2DM patients was conducted. PubMed, Embase, EBSCO, Web of Science, and China National Knowledge Infrastructure were systematically searched for original articles published from January 2000 to July 2023. The effect size was expressed as the mean difference (MD) or standardized mean difference (SMD) and its 95% confidence interval (CI). Subgroup analyses were performed by exercise mode (high-intensity interval training [HIIT] or moderate-intensity continuous training [MICT]) and intervention duration (>6 or ≤6 months).

**Results:**

Compared to usual care, both HIIT and MICT significantly affected left ventricular end-diastolic volume (MD: 19.44, 95% CI: 13.72 to 25.17, *p* < 0.00001; I^2^ = 42%; MD: 13.90, 95% CI: 7.64 to 20.16, *p* < 0.0001; I^2^ = 0%), but only HIIT significantly affected left ventricular mass (MD: 17.04 g, 95% CI: 5.45 to 28.62, *p* = 0.004; I^2^ = 0%). HIIT significantly improved left ventricular ejection fraction (MD: 5.52, 95% CI: 2.31 to 8.73, *p* = 0.0008; I^2^ = 0%), as did MICT in the ≤6 months subgroup (MD: 1.36, 95% CI: 0.61 to 2.10, *p* = 0.0004; I^2^ = 0%). Neither significantly affected systolic tissue velocity. HIIT significantly improved VO_2_peak (MD: 8.04, 95% CI: 6.26 to 9.83, *p* < 0.00001; I^2^ = 0%), as did MICT in the ≤6 months subgroup (MD: 3.33, 95% CI: 2.39 to 4.27, *p* < 0.00001; I^2^ = 0%).

**Conclusion:**

Exercise significantly improved cardiac structure, systolic function, and VO_2_peak, but did not significantly affect diastolic function in T2DM patients. HIIT seemed to be superior to MICT at improving VO_2_peak and left ventricular ejection fraction in T2DM patients.

**Systematic Review Registration:** https://www.crd.york.ac.uk/PROSPERO/, PROSPERO registration no.: CRD4242018087376

## 1 Background

Type 2 diabetes mellitus (T2DM) patients have an increased risk of heart failure (HF), up to 2–5 fold, compared to age-matched controls (Segar et al., 2021; Kannel et al., 1974; Nichols et al., 2004). Even after adjusting for other risk factors, such as age, hypertension, hypercholesterolemia, and coronary artery disease, the incidence of HF among T2DM patients remains high (Kannel et al., 1974; Nichols et al., 2004; Gulsin et al., 2018). Thus, the term “diabetic cardiomyopathy” was proposed and was initially used to describe ventricular dysfunction in the absence of coronary artery disease and hypertension in T2DM patients (Adeghate and Singh, 2014; Ritchie and Abel, 2020; Dillmann, 2019). Diabetic cardiomyopathy seems to worsen with time (Salzano et al., 2022; Jankauskas et al., 2021). The initial stage is characterized by left ventricular hypertrophy, increased atrial filling pressure, and diastolic dysfunction accompanied by HF with preserved ejection fraction (HFpEF); this is followed by systolic dysfunction accompanied by HF with reduced ejection fraction (HFrEF). Young T2DM patients with a short disease course (mean age 32 years) exhibit subclinical diastolic impairment and changes in their cardiac anatomical and functional parameters (Khan et al., 2014). Once HF occurs, it is linked to an unfavorable prognosis. Therefore, it is crucial to identify treatments that can alter the course of T2DM and prevent cardiac comorbidity.

Exercise is well established as a powerful tool to reduce blood glucose levels and improve cardiorespiratory fitness, and its effects on the prognosis (including all-cause death, cardiovascular death, and HF hospitalization) of T2DM have been well demonstrated (Marwick et al., 2009; Arrieta-Leandro et al., 2023; Wing et al., 2013; Colberg et al., 2010). Based on clinical evidence, the latest guidelines strongly recommend exercise as a management option for T2DM and HF patients (Jingming et al., 2023; Junbo et al., 2022; Kemps et al., 2019; Virani et al., 2023). However, the mechanisms and intermediate links underlying the effects of exercise on the heart in T2DM remain to be clarified. The results of clinical studies assessing the effects of exercise on cardiac structure and function in T2DM have been inconsistent. A study by Schrauwen-Hinderling et al. (2011) showed that 12 weeks of exercise improved left ventricular ejection fraction (LVEF) in T2DM but not left ventricular end-diastolic volume (LVEDV). Several studies (Van Ryckeghem et al., 2022; Jonker et al., 2013; Cadeddu et al., 2016) reported that exercise did not improve cardiac function in T2DM. Studies on exercise mode revealed the benefits of high-intensity interval training (HIIT) on cardiac structure (Cassidy et al., 2016) and systolic (Cassidy et al., 2016; Hollekim-Strand et al., 2014) and diastolic (Cassidy et al., 2016; Hollekim-Strand et al., 2014) function, while moderate-intensity continuous training (MICT) improved diastolic but not systolic function (Hollekim-Strand et al., 2014).

It remains unclear which exercise parameters (intensity, type, duration, etc.,) improve cardiac function and reverse adverse remodeling. Therefore, this meta-analysis was conducted to clarify the effects of different exercise modes on cardiac structure, cardiac function, and VO_2_peak in T2DM patients. Subgroup analyses by exercise mode (HIIT or MICT) and duration (>6 or ≤6 months) were performed.

## 2 Methods

### 2.1 Data sources

PubMed, Embase, EBSCO, Web of Science, and China National Knowledge Infrastructure were systematically searched for original articles published from January 2020 to September 2023 using the following keywords: Exercise (OR Physical Activity OR Exercise Intervention OR Training) AND Diabetes Mellitus, Type 2 (OR Type 2 Diabetes Mellitus OR Type 2 Diabetes) AND Cardiac Function (OR Diastolic OR Systolic OR Cardiac OR Heart).

### 2.2 Study reporting and eligibility criteria

This meta-analysis is reported following the Preferred Reporting Items for Systematic Reviews and Meta-Analyses (PRISMA) guidelines (Moher et al., 2009; Page et al., 2021). The protocol was registered with PROSPERO (ref. CRD42018087376).

The inclusion criteria were as follows: (1) type of studies: published randomized controlled trials (RCTs) and nonrandomized controlled trial in Chinese or English; (2) participants: adult T2DM patients (≥18 years); (3) intervention: exercise, with detailed descriptions of exercise regimen (4) comparator: usual care (i.e., intervention that any patient would have received in the framework of T2DM management); with no exercise intervention; (5) primary outcome measure: included at least one cardiac function parameter; and (6) study duration: exercise intervention period ≥8 weeks.

The exclusion criteria were as follows: (1) studies using animal models, conference abstracts, book chapters, reviews, or unpublished articles; (2) outcome measures did not meet the inclusion requirements; (3) exercise intervention was combined with diet intervention or other lifestyle changes; (4) lack of required control group (usual care); (5) repeat publications; and (6) study did not meet the inclusion criteria.

### 2.3 Data extraction

The extracted data included (1) general information (first author, publication year, sample size, and mean age of each group); (2) intervention information (duration, exercise type and site, and session length, intensity, and frequency); (3) cardiac imaging method; and (4) outcomes.

### 2.4 Outcome measures

The outcome measures were (1) cardiac anatomic changes, including left ventricular mass (LVM), left ventricular mass index (LVMI), left ventricular end-diastolic volume (LVEDV), and left ventricular end-diastolic diameter (LVEDD); (2) cardiac systolic function changes, including left ventricular ejection fraction (LVEF) and systolic tissue velocity (S); (3) cardiac diastolic function changes, including peak early diastolic mitral inflow velocity (E), peak late diastolic mitral inflow velocity (A), ratio of peak early to late diastolic mitral inflow velocity (E/A), mitral inflow to mitral relaxation velocity ratio (E/eʹ), and E-wave deceleration time (Dt); and (4) changes in VO_2_peak.

### 2.5 Quality assessment of included studies

Methodological quality was assessed by two investigators (H-JH and Z-D) using the Physiotherapy Evidence Database (PEDro) scale. This includes eligibility criteria, random allocation, concealed allocation, group similarity at baseline, subject blinding, therapist blinding, assessor blinding, dropouts ≤15%, intention-to-treat analysis, between-group comparisons, and point and variability measures. Methodological quality was evaluated based on manually calculated PEDro scores, with >5, 4–5, and 0–3 representing high (Sherrington et al., 2000), moderate, and low quality, respectively.

Risk of bias was assed using the Cochrane Collaboration’s tool (Higgins et al., 2011). This includes random sequence generation, allocation concealment, blinding of participants and personnel, blinding of outcome assessment, incomplete outcome data, selective reporting, and other sources of bias. For each study, the risk of bias was reported as low, unclear, or high risk. Studies with >2 or >4 high-risk components were considered to have moderate and high risk of bias, respectively.

The certainty of the evidence for each outcome was evaluated using the Grading Recommendations Assessment, Development and Evaluation (GRADE) approach. The Guideline Development Tool (https://www.Gradepro.org) was used to formulate the evidence profile table.

Any inconsistencies between the two investigators (H-JH and Z-D) were discussed with a third investigator (M-FR) until a consensus was reached.^1^

### 2.6 Statistical analysis

All the variables of interest were continuous and are expressed as mean ± SD. The effects of exercise on the outcomes were compared between the intervention and control groups. If the same outcome measurement scale was used in different studies, the pooled results were expressed as mean difference (MD) and 95% confidence interval (CI); otherwise, the results were expressed as standardized mean difference (SMD) and 95% CI. A two-sided *p* < 0.05 was considered significant. The heterogeneity of the results was assessed using I^2^ statistics.

The sensitivity analyses involved using the leave-one-out method, with SMD being used to assess the effect size. Subgroup analyses by exercise mode (HIIT or MICT) were to be conducted if each subgroup contained ≥2 studies. Subgroup analyses by exercise intervention duration (>6 or ≤6 months) were also to be conducted if each subgroup contained ≥2 studies.

All analyses were performed using Review Manager v5.4 and STATA v17.0.

## 3 Results

### 3.1 Search results and baseline characteristics

**FIGURE 1:**
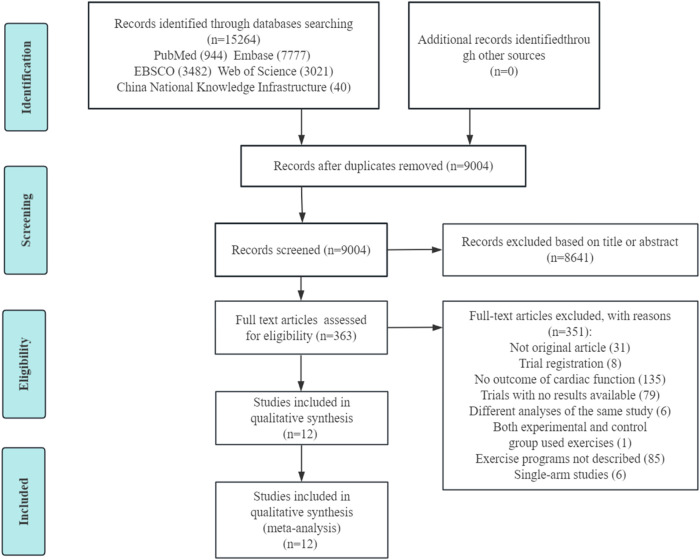
PRISMA flow diagram of study selection.

The literature search results and assessment process are shown in Figure 1. Of the 15,264 articles identified by the literature search, 351 underwent full-text assessment after screening the titles and/or abstracts. Of these, 12 articles (Cassidy et al., 2016; Jianghua, 2022; Gulsin et al., 2020a; Hare et al., 2011; Hordern et al., 2009; Wilson et al., 2019; Schmidt et al., 2013; Brassard et al., 2007; Weiwei et al., 2019; Sacre et al., 2014; McGavock et al., 2004; Loimaala et al., 2007) were included in this meta-analysis. Two articles (Jianghua, 2022; Weiwei et al., 2019) had two different exercise protocols along with a non-exercise control group. This provided two independent comparisons in each article, so 14 exercise arms with 14 sets of data were finally included, and the sample size of the control group was halved or split into (n-1)/2 and (n+1)/2 for the two abovementioned articles (Jianghua, 2022; Weiwei et al., 2019).

Among the 12 studies, a total of 714 participants were enrolled, comprising 367 in the exercise groups and 347 in the control groups. The mean age ranged from 48 to 60 years old. The exercise intervention durations ranged from 10 weeks to 3 years, with the majority having a duration of 3 months, only one study (McGavock et al., 2004) having a duration of <3 months, and three studies (Hare et al., 2011; Hordern et al., 2009; Loimaala et al., 2007) having a duration of >1 year. The exercise session frequency ranged from 3 to 5 times/week. The exercise session length ranged from 20 to 60 min. Among the 14 exercise arms, there were 11 MICT groups and 3 HIIT groups. The main characteristics of the participants and exercise interventions are presented in Table 1.

**TABLE 1:** Baseline characteristics of eligible studies.

Article first author and year	Participant characteristics	Exercise intervention				Measurements	Cardiorespiratory fitness	Cardiac structure and function indices
		Exercise program type and site	Session length and intensity	Duration and frequency	Exercise mode			
Cassidy et al. (2016)	Asymptomatic T2DM Exercise: n = 12; age 59 ± 9 y Usual care: n = 11; age 61 ± 9 y	Aerobic training (supervised)	40 min; 16∼17 RPE 5 × 2–3:50 (16∼17RPE) interval 3 min	12 weeks; 3/week	HIIT	MRI	VO_2Peak_	LVEF, LVM, LVEDV
Jianghua (2022)	Asymptomatic T2DM Exercise1: n = 16; age 54.8 ± 6.0 y Exercise2: n = 15; age56.5 ± 6.8 y Usual care: n = 16; age 55.5 ± 7.4 y	Aerobic and resistance training (supervised)	1: 60 min; 4 × 4 min (70–90%HRmax) interval 3 min (<50%HRmax) 2: 60 min; 30 min (55–70%HRmax)	12 weeks; 3/week	HIIT	Echo	VO_2Peak_	LVEF, LVEDD, LVM
					MICT			
Gulsin et al. (2020a)	Asymptomatic T2DM Exercise: n = 22; age 50.1 ± 7.3 y Usual care: n = 30; age50.1 ± 6.1 y	Aerobic training (supervised)	50 min; ∼60%VO_2_peak	12 weeks; 3/week	MICT	Echo CMR	VO_2Peak_	LVEF, LVM, LVEDV,E/A, E/e
Hare et al. (2011)	T2DM Exercise: n = 94; age 56 ± 11 y Usual care: n = 92; age 55 ± 8 y	Aerobic and resistance training Initial 4 weeks (supervised) After 4 weeks (unsupervised)	60 min; 12–13 RPE	3 years; 2/week	MICT	Echo	VO_2Peak_	LVEF, LVMI, E
Hordern et al. (2009)	T2DM Exercise: n = 111; age 56.1 ± 11.7 y Usual care: n = 112; age 55 ± 8.5 y	Aerobic and resistance training Initial 4 weeks (supervised) After 4 weeks (unsupervised)	60 min; 12–13 RPE	1 year; 2/week	MICT	Echo	VO_2Peak_	LVEF, LVM, LVEDD, E, S
Wilson et al. (2019)	Asymptomatic T2DM Exercise: n = 11; age 52 ± 2 y Usual care: n = 5; age 51 ± 5 y	Aerobic training (supervised)	20 min; Month1:10 × 1 min (>90%HRpeak) interval 1 min Month2:5 × 2 min (>90%HRpeak) interval 2 min Month3:4 × 3 min (>90%HRpeak) interval 2 min	3 months; 3/week	HIIT	Echo	VO_2Peak_	LVEF, LVMI, LVEDV, E, A, E/A, E/e, S
Schmidt et al. (2013)	Asymptomatic T2DM Exercise: n = 7; age 50.7 ± 7.1 y Usual care: n = 9; age 48.7 ± 9.2 y	Aerobic training (supervised)	60 min; 5 × 10 min (76–88% HRmax) interval 2 min	12 weeks; 2/week	MICT	Echo	VO_2Peak_	LVEF, LVMI, LVEDD, E, A, E/A, E/e, Dt, S
Brassard et al. (2007)	T2DM and LVDD Exercise: n = 11; age 58 ± 5 y Usual care: n = 9; age 57 ± 6 y	Aerobic training (supervised)	60 min; 60–70% VO_2_max	12 weeks; 3/week	MICT	Echo	VO_2Peak_	LVEF, LVM, LVMI, LVEDD, E, A, E/A, Dt
Weiwei et al. (2019)	Asymptomatic T2DM Exercise1: n = 9; age 55.2 ± 7.6 y Exercise2: n = 7; age 52.7 ± 6 y Usual care: n = 10; age 52.6 ± 6.6 y	1: Aerobic training 2: Aerobic and resistance training	1: 60 min; 60–80%HRmax 2: 60 min; 60–80%HRmax	6 months; 6/week	MICT	Echo	—	LVEF, E, A, E/A
					MICT			
Sacre et al. (2014)	Asymptomatic T2DM Exercise: n = 24; age 59 ± 10 y Usual care: n = 25; age 60 ± 9 y	Aerobic and resistance training Initial 4 weeks (supervised) After 4 weeks (unsupervised)	75; 20–40 min12∼13 RPE	6 months; 3/week	MICT	Echo	VO_2Peak_	LVEF, LVMI, E, A, E/A, E/e, Dt, S
McGavock et al. (2004)	Asymptomatic T2DM Exercise: n = 11; age 58 ± 7 y Usual care: n = 7; age 59 ± 5 y	Aerobic and resistance training (supervised)	—; 30–55 min 65–75%HRreserve 3sets 10–15rp: 50–65% 1RM	10 weeks; 3/week	MICT	Echo	VO_2Peak_	E, A, E/A, Dt
Loimaala et al. (2007)	Asymptomatic T2DM Exercise: n = 24; age 52.8 ± 6 y Usual care: n = 24; age 52.8 ± 5.2 y	Aerobic and resistance training	30 min; 65–75% VO2max 3sets 10–12rp at 70–80% 1RM	12 months; 2/week	MICT	Echo	VO_2Peak_	LVEF, LVEDD, E, A,

LVM, left ventricular mass; LVMI, left ventricular mass indexed; LVEDV, left ventricular end-diastolic volume; LVEDD, left ventricular end-diastolic diameter; LVEF, left ventricular ejection fraction; S systolic tissue velocity; E peak early diastolic mitral inflow velocities; A peak late diastolic mitral inflow velocities; E/A the ratio of peak early to late diastolic mitral inflow velocities; E/e the mitral inflow to mitral relaxation velocity ratio; Dt the E-wave deceleration time; VO_2_peak peak oxygen uptake; Echo echocardiograph; MRI, magnetic resonance imaging; CMR, cardiac magnetic resonance; T2DM, type 2 diabetes mellitus; LVDD, left ventricular diastolic dysfunction.

Table 2 shows the main results of the included studies. LVM, LVMI, LVEDV, LVEDD, LVEF, E, A, S, Dt, E/A ratio, E/e’ ratio, and VO_2_peak were reported in 5 (Cassidy et al., 2016; Jianghua, 2022; Gulsin et al., 2020a; Hordern et al., 2009; Wilson et al., 2019), 6 (Gulsin et al., 2020a; Hare et al., 2011; Hordern et al., 2009; Schmidt et al., 2013; Brassard et al., 2007; Sacre et al., 2014), 4 (Cassidy et al., 2016; Gulsin et al., 2020a; Wilson et al., 2019; Schmidt et al., 2013), 4 (Jianghua, 2022; Hordern et al., 2009; Schmidt et al., 2013; Brassard et al., 2007), 10 (Cassidy et al., 2016; Jianghua, 2022; Gulsin et al., 2020a; Hare et al., 2011; Hordern et al., 2009; Wilson et al., 2019; Schmidt et al., 2013; Brassard et al., 2007; Weiwei et al., 2019; Sacre et al., 2014), 8 (Hordern et al., 2009; Wilson et al., 2019; Schmidt et al., 2013; Brassard et al., 2007; Weiwei et al., 2019; Sacre et al., 2014; McGavock et al., 2004; Loimaala et al., 2007), 6 (Wilson et al., 2019; Schmidt et al., 2013; Brassard et al., 2007; Weiwei et al., 2019; McGavock et al., 2004; Loimaala et al., 2007), 4 (Hordern et al., 2009; Wilson et al., 2019; Schmidt et al., 2013; Sacre et al., 2014), 4 (Schmidt et al., 2013; Brassard et al., 2007; Sacre et al., 2014; McGavock et al., 2004), 8 (Gulsin et al., 2020a; Wilson et al., 2019; Schmidt et al., 2013; Brassard et al., 2007; Weiwei et al., 2019; Sacre et al., 2014; McGavock et al., 2004; Loimaala et al., 2007), 4 (Gulsin et al., 2020a; Wilson et al., 2019; Schmidt et al., 2013; Sacre et al., 2014) and 10 (Jianghua, 2022; Gulsin et al., 2020a; Hare et al., 2011; Hordern et al., 2009; Wilson et al., 2019; Schmidt et al., 2013; Brassard et al., 2007; Sacre et al., 2014; McGavock et al., 2004; Loimaala et al., 2007) studies, respectively. Cardiac structure and function were evaluated by magnetic resonance imaging in 1 study (Cassidy et al., 2016), echocardiography in 10 studies (Jianghua, 2022; Hare et al., 2011; Hordern et al., 2009; Wilson et al., 2019; Schmidt et al., 2013; Brassard et al., 2007; Weiwei et al., 2019; Sacre et al., 2014; McGavock et al., 2004; Loimaala et al., 2007), and cardiac magnetic resonance in 1 study (Gulsin et al., 2020a).

**TABLE 2:** The outcome changes of eligible studies.

References	LVM	LVMI	LVEDV	LVEDD	LVEF	E	A	S	Dt	E/A	E/e	VO_2_peak
Cassidy et al. (2016)	104/116	NR	118/126	NR	65/70	NR	NR	NR	NR	NR	NR	NR
Jianghua (2022)	110.5/129.8	NR	NR	3.86/4	66.7/70.5	NR	NR	NR	NR	NR	NR	20.9/27.4
	101.3/111		NR	3.57/3.69	64.3/67.1							19.2/22.4
Gulsin et al. (2020a)	123.1/122	57/56.9	147.2/145.1	NR	66.8/66	NR	NR	NR	NR	0.94/1	8.6/8.6	17.2/18.2
Hare et al. (2011)	NR	85.5/88.4	NR	NR	66/64.6	NR	NR	NR	NR	NR	NR	21.5/24.7
Hordern et al. (2009)	203.3/201.5	96.7/96.4	NR	4.7/4.8	70.9/72.8	5.5/6.1	NR	5.3/6	NR	NR	NR	21.9/24.2
Wilson et al. (2019)	204.5/205.8	NR	113.5/125	NR	59/60	63/68	70/71	8/8	NR	0.93/0.95	8.5/9.9	24.1/27.6
Schmidt et al. (2013)	NR	79/91.4	119.2/135	5/5.3	58.1/59.5	0.07/0.09	0.11/0.11	6.1/6.9	214.8/186	0.9/1.1	9.7/8.4	30.5/34.1
Brassard et al. (2007)	NR	88/81	NR	4.9/5	66/67	60/62	63/56	NR	209/223	0.76/0.96	NR	28.6/32.7
Weiwei et al. (2019)	NR	NR	NR	NR	60.4/61.3	52.2/54.9	62.8/53.9	NR	NR	0.83/1.03	NR	NR
	NR	NR			60.9/61.8	53.6/56.2	65.3/56.4			0.82/0.99		
Sacre et al. (2014)	NR	79/86	NR	NR	62/59	4.7/5.8	NR	5.3/5.7	232/213	0.89/0.94	13.4/15.9	26.8/29.8
McGavock et al. (2004)	NR	NR	NR	NR	NR	87/88	95/92	NR	256/253	1/1	NR	21.3/24.5
Loimaala et al. (2007)	NR	NR	NR	NR	NR	0.64/0.7	0.68/0.7	NR	NR	0.94/1	NR	32/34.7

Exercise-pre/Exercise-post; LVM, left ventricular mass; LVMI, left ventricular mass indexed; LVEDV, left ventricular end-diastolic volume; LVEDD, left ventricular end-diastolic diameter; LVEF, left ventricular ejection fraction; S systolic tissue velocity; E peak early diastolic mitral inflow velocities; A peak late diastolic mitral inflow velocities; E/A the ratio of peak early to late diastolic mitral inflow velocities; E/e the mitral inflow to mitral relaxation velocity ratio; Dt the E-wave deceleration time; VO2peak peak oxygen uptake; NR, not report.

### 3.2 Quality assessment and risk of bias in individual studies

Quality assessments of the 12 studies are shown in Table 3. Of the 12 studies, 8 were considered high quality, 2 moderate quality, and 2 low quality, which was mainly driven by the lack of blinded designs in any study plus the nonrandom design in the studies by Sacre et al. (2014) and Schmidt et al. (2013).

**TABLE 3:** Quality assessment of eligible studies.

References	Allocation of randomization	Concealed allocation	Similarity baseline	Subject blinding	Therapist blinding	Assessor blinding	Point and variability measures	Dropouts≤15%	Between-group comparisons	Intention-to-treat analysis	Total PEDro score
Cassidy et al. (2016)	1	1	1	0	0	0	1	1	1	1	7
Jianghua (2022)	1	1	0	0	0	1	1	1	1	1	7
Gulsin et al. (2020a)	1	1	1	0	0	0	1	1	1	1	7
Hare et al. (2011)	1	1	0	0	0	0	1	0	1	1	5
Hordern et al. (2009)	1	1	0	0	0	0	1	0	1	1	5
Wilson et al. (2019)	1	1	1	0	0	0	1	1	1	1	7
Schmidt et al. (2013)	0	0	0	0	0	0	1	0	1	1	3
Brassard et al. (2007)	1	1	1	0	0	0	1	1	1	1	7
Weiwei et al. (2019)	1	1	1	0	0	0	1	1	1	1	7
Sacre et al. (2014)	0	0	0	0	0	0	1	1	1	0	3
McGavock et al. (2004)	1	1	1	0	0	0	1	0	1	1	6
Loimaala et al. (2007)	1	1	0	0	0	0	1	1	1	1	6

The overall risk of bias was low in most studies. The details are presented in the Additional material (Additional file: Supplementary Figure 6).

### 3.3 Results of the main and subgroup analyses

#### 3.3.1 Effects of exercise on LV dimensions and structure

**FIGURE 2:**
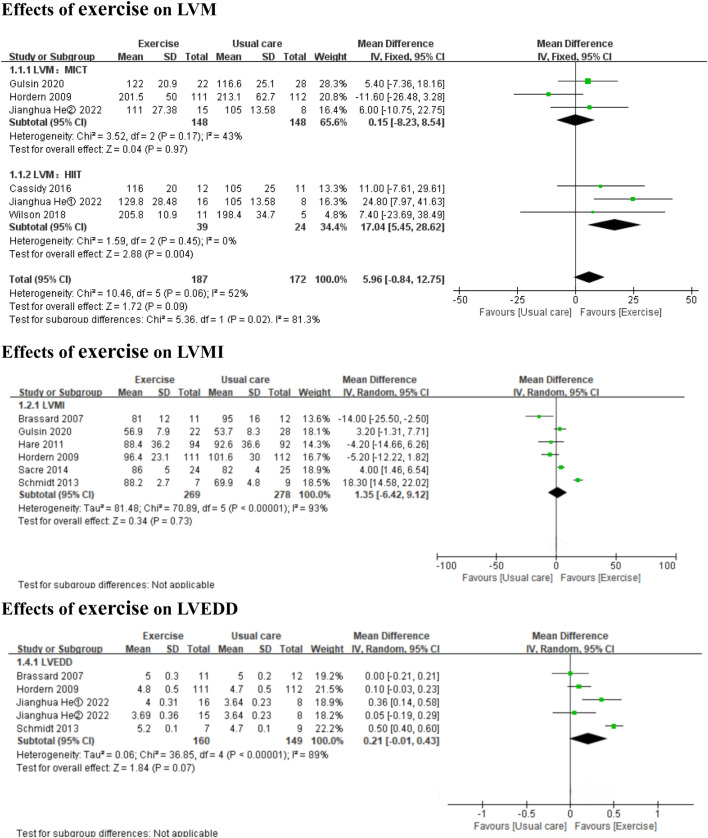
Forest plots of the effects of exercise on LVM, LVMI, and LVEDD. LVM left ventricular mass; LVMI left ventricular mass indexed; LVEDD left ventricular end-diastolic diameter.

**FIGURE 3:**
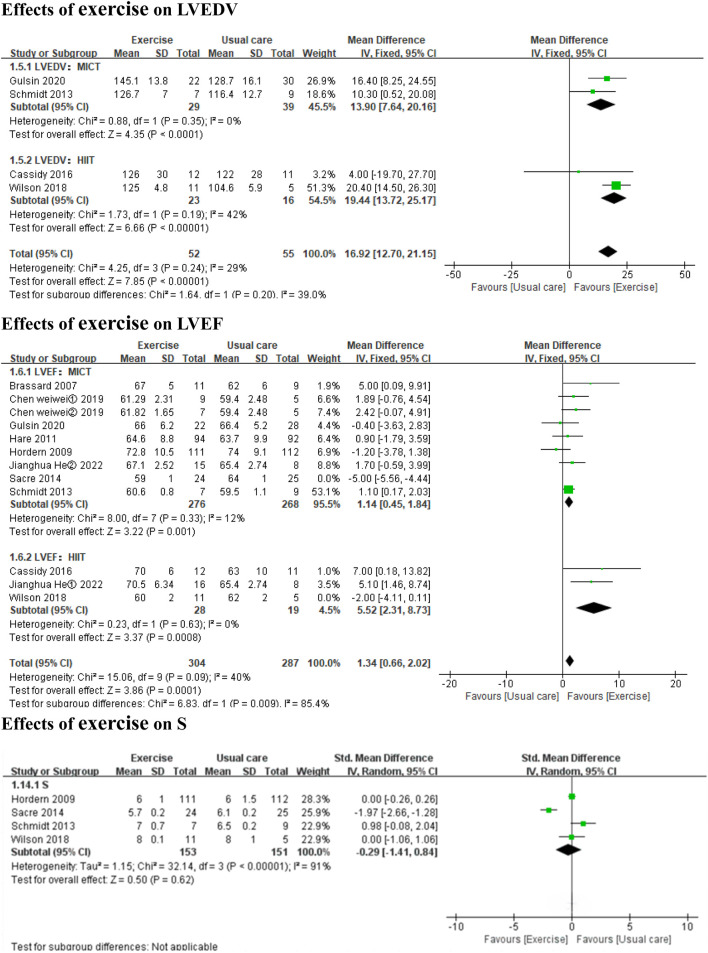
Forest plots of the effects of exercise on LVEDV, LVEF, and S. LVEDV left ventricular end-diastolic volume; LVEF left ventricular ejection fraction; S systolic tissue velocity.

Compared to usual care, HIIT significantly improved LVM (*3 HIIT studies,* MD: 17.04 g, 95% CI: 5.45 to 28.62, *p* = 0.004; I^2^ = 0%), but MICT did not have a significant effect (*3 MICT studies,* MD: 0.15 g, 95% CI: −8.23 to 8.54, *p* = 0.97; I^2^ = 43%) (Figure 2). Exercise did not significantly affect LVMI or LVEDD (*6 MICT studies*, MD: 1.35, 95% CI: −6.24 to 9.12, *p* = 0.73; I^2^ = 93%; *4 MICT studies and 1 HIIT studies,* MD: 0.21cm, 95% CI: −0.01 to 0.43, *p =* 0.07; I^2^ = 89%) (Figure 2). Both HIIT and MICT significantly affected LVEDV (*2 HIIT studies,* MD: 19.44 mL, 95% CI: 13.72 to 25.17, *p* < 0.00001; I^2^ = 42%; *2 MICT studies,* MD: 13.90 mL, 95% CI: 7.64 to 20.16, *p* < 0.0001; I^2^ = 0%) (Figure 3).

In the subgroup analyses by intervention duration, MICT did not significantly affect LVMI in either the ≤6 months subgroup (*4 MICT studies,* MD: 4.09, 95% CI: −5.22 to 13.40, *p* = 0.39; I^2^ = 95%) or the >6 months subgroup (*2 MICT studies,* MD: −4.89, 95% CI: −10.72 to 0.94, *p* = 0.10; I^2^ = 0%) (Additional file: Supplementary Figure 1). No subgroup analyses by intervention duration were conducted for LVM (**only 3 MICT studies**), LVEDD (**only 1 MICT study >6** **months**), or LVEDV (**only 2 MICT studies**), as these subgroup analyses were to be performed only if each subgroup contained ≥2 studies.

#### 3.3.2 Effects of exercise on systolic function

Both HIIT and MICT significantly improved LVEF (*3 HIIT studies,* MD: 5.52, 95% CI: 2.31 to 8.73, *p* = 0.0008; I^2^ = 0%; *8 MICT studies,* MD: 1.14, 95% CI: 0.45 to 1.84, *p* = 0.001; I^2^ = 12%) (Figure 3). Exercise did not significantly affect S (*3 MICT studies and 1 HIIT study*; SMD: −0.29, 95% CI: −1.41 to 0.84, *p* = 0.62; I^2^ = 91%) (Figure 3).

In the subgroup analyses by intervention duration, MICT significantly improved LVEF in the ≤6 months subgroup (*6 MICT studies,* MD: 1.36, 95% CI: 0.61 to 2.10, *p* = 0.0004; I^2^ = 0%), but the result was non-significant in the >6 months subgroup (*2 MICT studies,* MD: −0.20, 95% CI: −2.06 to 1.67, *p* = 0.84; I^2^ = 18%) (Additional file: Supplementary Figure 2). No subgroup analysis by intervention duration was conducted for S (**3 MICT studies and 1 HIIT study**).

#### 3.3.3 Effects of exercise on diastolic function

**FIGURE 4:**
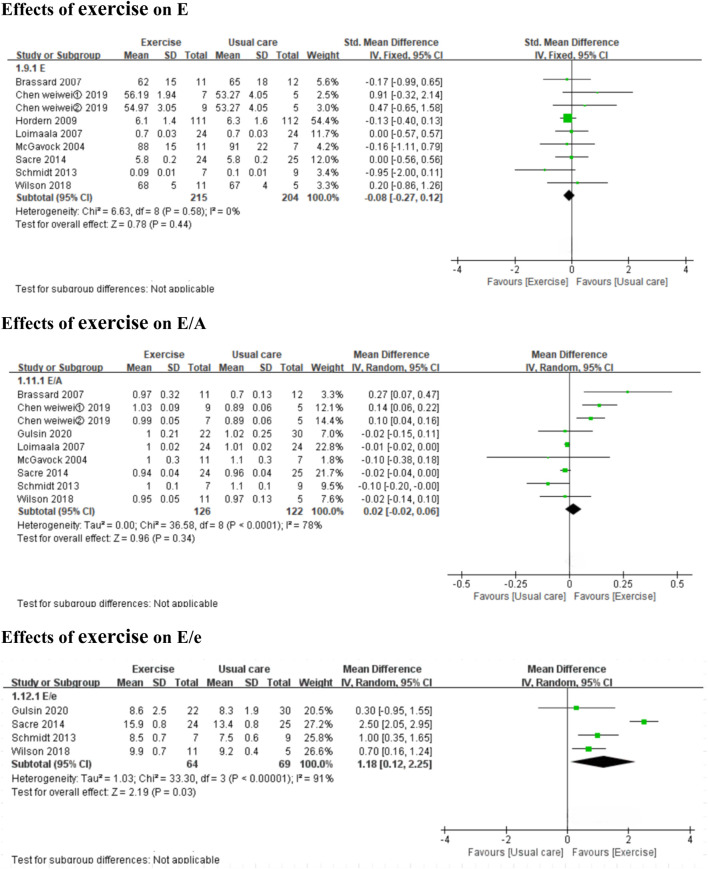
Forest plots of the effects of exercise on E, E/A, and E/e. E peak early diastolic mitral inflow velocities; E/A the ratio of peak early to late diastolic mitral inflow velocities; E/e the mitral inflow to mitral relaxation velocity ratio.

**FIGURE 5:**
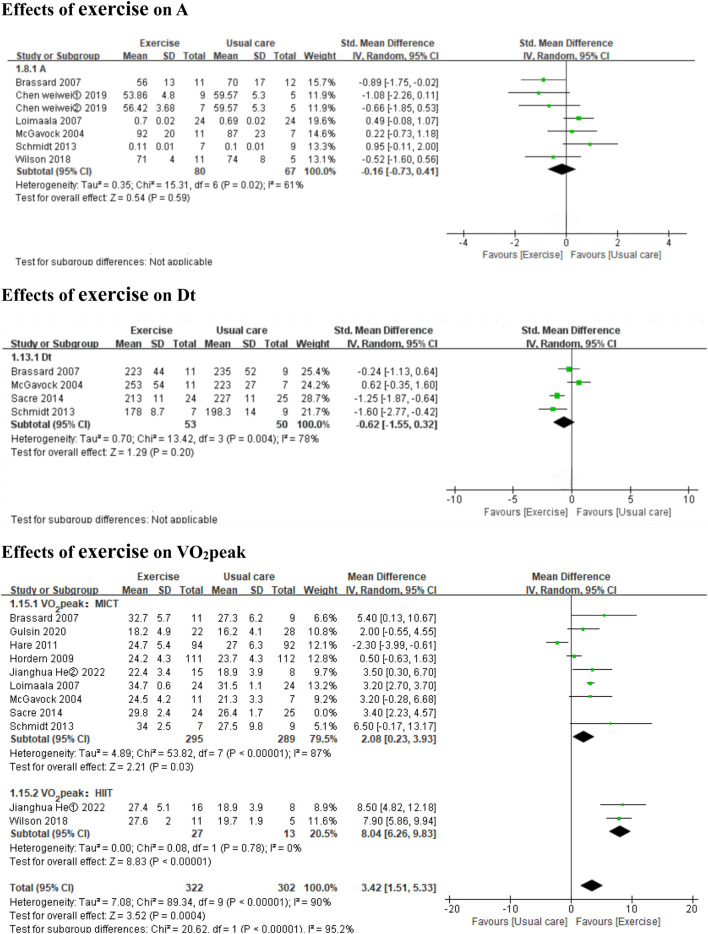
Forest plots of the effects of exercise on A, Dt, and VO_2_peak. A peak late diastolic mitral inflow velocities; Dt the E-wave deceleration time; VO2peak peak oxygen uptake.

Exercise improved the E/e’ ratio (*3 MICT studies and 1 HIIT studies,* MD: 1.20, 95% CI: 0.37 to 2.03, *p =* 0.005; I^2^ = 88%) (Figure 4), but not E, A, Dt, or the E/A ratio (*7 MICT studies and 1 HIIT studies,* SMD: −0.08, 95% CI: −0.27 to 0.12, *p* = 0.44; I^2^ = 0%; *5 MICT studies and 1 HIIT studies,* SMD: −0.16, 95% CI: −0.73 to 0.41, *p* = 0.59; I^2^ = 61%; *4 MICT studies,* MD: −0.62 m, 95% CI: −1.55 to 0.32, *p* = 0.20; I^2^ = 78%; *7 MICT studies and 1 HIIT studies,* MD: 0.02, 95% CI: −0.02 to 0.06, *p* = 0.34; I^2^ = 78%) (Figures 4, 5).

In the subgroup analyses by intervention duration, MICT did not significantly affect E in the ≤6 months subgroup (*5 MICT studies*, SMD: −0.04, 95% CI: −0.39 to 0.31, *p* = 0.83; I^2^ = 18%) or the >6 months subgroup (*2 MICT studies*, SMD: −0.11, 95% CI: −0.35 to 0.13, *p* = 0.37; I^2^ = 0%) (Additional file: Supplementary Figure 3). No subgroup analyses by intervention duration were conducted for E/e’ and Dt (**0 studies ≤6** **months**) or A and E/A (**only 1 MICT study >6** **months**).

#### 3.3.4 Effects of exercise on cardiopulmonary function

Both HIIT and MICT significantly improved VO_2_peak (*3 HIIT studies,* MD: 8.04 mL kg^−1^ min^−1^, 95% CI: 6.26 to 9.83, *p* < 0.00001; I^2^ = 0%; *9 MICT studies,* MD: 2.08 mL kg^−1^ min^−1^, 95% CI: 0.23 to 3.93, *p* = 0.03; I^2^ = 87%) (Figure 5).

In the subgroup analyses by intervention duration, MICT significantly improved VO_2_peak in the ≤6 months subgroup (*6 MICT studies,* MD: 3.33 mL kg^−1^ min^−1^, 95% CI: 2.39 to 4.27, *p* < 0.00001; I^2^ = 0%), but the result was non-significant in the >6 months subgroup (*3 MICT studies,* MD: 0.55 mL kg^−1^ min^-1^, 95% CI: −2.46 to 3.57, *p* = 0.72; I^2^ = 96%) (Additional file: Supplementary Figure 4).

### 3.4 Sensitivity analyses

To assess the robustness of the effects, sensitivity analyses were performed by using SMD for LVM, LVEDV, LVEF, and VO_2_peak. The effects sizes for the effects of HIIT on LVM, LVEF, and VO_2_peak were consistent with the main analysis (LVM, SMD: 0.61, 95% CI: 0.08 to 1.14, *p* = 0.02; I^2=^0%; LVEF, SMD: 0.86, 95% CI: 0.24 to 1.48, *p* = 0.006; I^2=^0%; VO_2_peak, SMD: 2.61, 95% CI: 0.61 to 4.60, *p* = 0.01; I^2=^73%), but non-significant for LVEDV (SMD: 0.74, 95% CI: −0.01 to 1.48, *p* = 0.05; I^2=^92%). The effect sizes for the effects of MICT on LVEDV and VO_2_peak were consistent with the main analysis, but non-significant for LVEF (Additional file: Supplementary Figure 5).

According to the GRADE evidence profile (Table 4), the certainty of the evidence was moderate or low for most of the outcomes, except for the effect of HIIT on LVEF and VO_2_peak, which had high certainty. Low certainty was mostly driven by the high risk of bias and inconsistency.

**TABLE 4:** GRADE evidence profile.

Certainty assessment							No of patients		Effect		Certainty	Importance
No of studies	Study design	Risk of bias	Inconsistency	Indirectness	Imprecision	Other considerations	[Exercise]	[Usual care]	Relative (95% CI)	Absolute (95% CI)		
**LVM:MICT** 3	randomised trials	not serious	not serious	not serious	serious	none	148	148	-	MD **0.15 higher** (8.23 lower to 8.54 higher)	⨁⨁⨁◯ Moderate	IMPORTANT
**LVM: HIIT** 3	randomised trials	not serious	not serious	not serious	serious	none	39	24	-	MD **17.04 higher** (5.45 higher to 28.62 higher)	⨁⨁⨁◯ Moderate	IMPORTANT
**LVMI** 6	randomised trials	serious	not serious	not serious	serious	none	269	278	-	MD **1.35 higher** (6.24 lower to 9.12 higher)	⨁⨁◯◯ Low	IMPORTANT
**LVEDD** 4	randomised trials	serious	not serious	not serious	serious	none	160	149	-	MD **0.21 higher** (0.01 lower to 0.43 higher)	⨁⨁◯◯ Low	IMPORTANT
**LVEDV:MICT** 2	randomised trials	very serious	not se rious	not serious	not serious	none	29	39	-	MD **13.9 higher** (7.64 higher to 20.16 higher)	⨁⨁◯◯ Low	IMPORTANT
**LVEDV: HIIT** 2	randomised trials	not serious	not serious	not serious	serious	none	23	16	-	MD **19.44 higher** (13.72 higher to 25.17 higher)	⨁⨁⨁◯ Moderate	IMPORTANT
**LVEF: MICT** 7	randomised trials	not serious	not serious	not serious	serious	none	276	268	-	MD **1.14 higher** (0.45 higher to 1.84 higher)	⨁⨁⨁◯ Moderate	IMPORTANT
**LVEF: HIIT** 2	randomised trials	not serious	not serious	not serious	not serious	none	28	19	-	MD **5.52 higher** (2.31 higher to 8.73 higher)	⨁⨁⨁⨁ High	IMPORTANT
**S** 4	randomised trials	serious	serious	not serious	serious	none	153	155	-	SMD **0.29 lower** (1.41 lower to 0.84 higher)	⨁◯◯◯ Very low	IMPORTANT
**E** 8	randomised trials	not serious	not serious	not serious	serious	none	215	204	-	SMD **0.08 lower** (0.27 lower to 0.12 higher)	⨁⨁⨁◯ Moderate	IMPORTANT
**E/A** 8	randomised trials	not serious	serious	not serious	serious	none	126	122	-	MD **0.02 higher** (0.02 lower to 0.06 higher)	⨁⨁◯◯ Low	IMPORTANT
**E/e** 4	randomised trials	serious	not serious	not serious	not serious	none	64	69	-	MD **1.18 higher** (0.12 higher to 2.25 higher)	⨁⨁⨁◯ Moderate	IMPORTANT
**A** 6	randomised trials	not serious	serious	not serious	serious	none	80	67	-	SMD **0.16 lower** (0.73 lower to 0.41 higher)	⨁⨁◯◯ Low	IMPORTANT
**Dt** 4	randomised trials	serious	serious	not serious	serious	none	53	50	-	SMD **0.62 lower** (1.55 lower to 0.32 higher)	⨁◯◯◯ Very low	IMPORTANT
**VO2peak: HIIT** 2	randomised trials	not serious	not serious	not serious	not serious	none	27	13	-	MD **8.04 higher** (6.26 higher to 9.83 higher)	⨁⨁⨁⨁ High	IMPORTANT
**VO2peak: MICT** 9	randomised trials	not serious	serious	not serious	serious	none	295	289	-	MD **2.08 higher** (0.23 higher to 3.93 higher)	⨁⨁◯◯Low	IMPORTANT

tricular mass; LVMI, left ventricular mass indexed; LVEDV, left ventricular end-diastolic volume; LVEDD, left ventricular end-diastolic diameter; LVEF, left ventricular ejection fraction; S systolic tissue velocity; E peak early diastolic mitral inflow velocities; A peak late diastolic mitral inflow velocities; E/A the ratio of peak early to late diastolic mitral inflow velocities; E/e the mitral inflow to mitral relaxation velocity ratio; Dt the E-wave deceleration time; VO2peak peak oxygen uptake.

Dt the E-wave deceleration time; VO_2_peak peak oxygen uptake; Echo echocardiograph; MRI, magnetic resonance imaging; CMR, cardiac magnetic resonance.

## 4 Discussion

This meta-analysis comprehensively and quantitively analyzed the effects of exercise on cardiac structure, cardiac function, and VO_2_peak in T2DM patients. The main findings included the following: (1) Both HIIT and MICT significantly affected LVEDV, and HIIT but not MICT significantly affected LVM. (2) Both HIIT and the ≤6 months MICT subgroup, but not the >6 months MICT subgroup, significantly improved LVEF, and exercise did not significantly affect S. (3) Exercise significantly affected the E/e’ ratio. (4) Both HIIT and MICT, but not the >6 months MICT subgroup, significantly improved VO_2_peak. Importantly, the findings of this meta-analysis suggest that HIIT is superior to MICT at improving VO_2_peak and LVEF in T2DM patients.

Left ventricular hypertrophy is common in T2DM and predicts adverse cardiovascular outcomes, including HF (Dawson et al., 2005; Jia et al., 2018). Reducing left ventricular hypertrophy can lower the associated cardiovascular risk (Koren et al., 2002). This meta-analysis showed that different exercise modes (HIIT vs. MICT) have different effects on LVM in T2DM. MICT significantly affected LVEDV, but it did not significantly affect LVM or LVMI, while HIIT significantly affected LVM and LVEDV. The HIIT-induced increase in LVM is known as “physiological hypertrophy” [a benign adaptive change that occurs after long-term exercise and does not cause cardiac fibrosis or cardiomyocyte apoptosis, but instead promotes left ventricular remodeling through a physiological response to growth signals (Frey et al., 2004)]. It should not be confused with “pathological hypertrophy” [characterized by collagen accumulation, increased wall thickness, myocyte hypertrophy and disarray as well as interstitial fibrosis, ultimately leading to cardiac dysfunction (Marian and Braunwald, 2017)], which is common in T2DM.

In clinical practice, left ventricular systolic function is primarily assessed by measuring LVEF, which is considered the gold standard for assessing this parameter despite some limitations (Lund et al., 2022). Its value is generally thought to be >50%. The main and subgroup analyses suggested that both HIIT and MICT significantly improved LVEF in T2DM patients, HIIT compared to usual care increased LVEF by 5.52% (95% CI: 2.31 to 8.73, I^2^ = 0%), while MICT compared to usual care increased LVEF by 1.14% (95% CI: 0.45 to 1.84, I^2^ = 12%). However, MICT for ≤6 months but not >6 months significantly improved LVEF, suggesting inadequate adherence by T2DM patients to long-term exercise. Although most of the included studies did not show impaired LVEF at baseline, the findings demonstrate the potential positive effects of exercise on systolic function. Exercise did not significantly affect S, unlike in a study by Hollekim-Strand et al. (2014), which showed that HIIT compared to MICT significantly improved S, which needs to be explored in future studies.

Diastolic dysfunction is the most commonly reported cardiac dysfunction induced by T2DM. T2DM patients with diastolic dysfunction compared to those with normal diastolic function face a 1.6–2.2 times higher risk of HF and death (From et al., 2010). The E/e’ and E/A ratios provide information about the diastolic filling pressure of the left ventricle. E/e’ ratios >15 are related to increased mean left ventricular diastolic filling pressure (obtained with ventricular catheters), while E/e’ ratios of 8–15 are unrelated (Nagueh et al., 2016; Ommen et al., 2000). The pooled result of this meta-analysis showed that exercise significantly affected the E/e’ ratio. However, the 4 included studies (Gulsin et al., 2020a; Wilson et al., 2019; Schmidt et al., 2013; Sacre et al., 2014) that reported on the effect of exercise on the E/e’ ratio showed that the baseline E/e’ ratios of patients with uncomplicated T2DM were within 8–14 [indicating normal E/e’ ratios, according to the recommendations from the American Society of Echocardiography and the European Association of Cardiovascular Imaging (Nagueh et al., 2016)]. E/A <1 has been interpreted as “diastolic dysfunction” in T2DM, and exercise (compared to usual care) did not improve E/A. The influence of exercise on diastolic function (E/e’ ratio, E/A ratio and Dt) remains unclear, and large trials are still needed. Notably, age should be taken into account when evaluating diastolic function using E/A (Lang et al., 2015; Caballero et al., 2015). Normal aging is associated with several cardiac changes, which may result in elderly individuals having filling patterns that resemble those observed in younger patients with mild diastolic dysfunction. In contrast, the E/e’ ratio is less age dependent (Nagueh et al., 2016; Caballero et al., 2015).

Decreased peak oxygen uptake (objectively measured by cardiopulmonary exercise testing) is common in T2DM (Nesti et al., 2021; Gulsin et al., 2020b). Exercise (compared to usual care) significantly improved cardiorespiratory fitness, based on VO_2_peak, HIIT induce higher increases in VO_2_peak than MICT according to the findings of this meta-analysis; HIIT compared to usual care increased VO_2_peak by 8.04 mL/kg/min (95% CI 6.26 to 9.83, I^2^ = 0%), while MICT compared to usual care increased VO_2_peak by 2.08 mL/kg/min (95% CI 0.23 to 3.93, I^2^ = 87%), which is consistent with the findings of previous meta-analyses (Liu et al., 2019; de Mello et al., 2022). A systemic analysis analyzing 13 studies by Liu et al. (2019). Showed that HIIT is more effective than continuous training in improving VO_2_peak by 3.37 mL/kg/min (95% CI 1.88 to 4.87, I^2^ = 48%). A more recent meta-analysis including 449 T2DM patients by de Mello et al. (2022). Revealed that HIIT yielded a significant increase VO_2_max by 5.09 mL/kg/min (95% CI 2.99 to 7.19, I^2^ = 80.89) *versus* the control. According to the Fick equation (Nesti et al., 2020), VO_2_peak is related to the rate of blood flow through the body (cardiac output) and the amount of oxygen extracted by the tissues as the blood flows from the arteries to the veins (△(a − v)O_2_). Therefore, the fact that HIIT increased LVEF and LVEDV may have translated into significantly greater improvements in VO_2_peak.

Despite the clinically meaningful improvements in cardiopulmonary function and cardiac structure and function outcomes by exercise, the underlying mechanisms required further investigation. The most likely mechanisms underlying exercise-induced cardiac benefits involve decreased LV cardiac fibrosis and inflammation as well as improved mitochondrial oxidative capacity (D’Haese et al., 2023; Lund et al., 2015; Kar et al., 2019; Cassidy et al., 2017), which are mainly based on animal studies. Decreased LV cardiac fibrosis improves myocardial systolic function and reduces left ventricular filling pressure. This could be the reason for the significant effects of exercise on LVEF, LVEDV, E/e’ ratio, and VO_2_peak in this meta-analysis. However, the increased LVM cannot be fully explained by this theory. Other mechanisms such as reducing plasma advanced glycation end-products (Hansen et al., 2013; Van den Eynde et al., 2020) and using more efficient energy sources (such as ketone bodies and fatty acids) instead of glucose (Anderson et al., 2009; van den Brom et al., 2009), may also be involved. Further research is required to illuminate the pathways underlying the cardiac effects of exercise in T2DM.

Two previous systematic and narrative reviews (Verboven et al., 2019; Anand et al., 2018) summarized completed and ongoing studies available on the same topic as ours. However, Verboven et al. (2019) conducted a narrative review instead of a meta-analysis, so the study lacked quantitative analyses. Furthermore, the meta-analysis by Anand et al. (2018) only included six nonrandomized trials, and several important recent studies were not included. The present meta-analysis included only intervention studies with usual care as the control group.

Although the present meta-analysis strictly followed the PRISMA guidelines, it has several limitations. First, in the subgroup analyses, the duration was stratified as >6 or ≤6 months, but most durations were 3 months. Second, heterogeneity in exercise interventions and study methods was not completely avoidable. Third, subgroup analyses by exercise type (endurance, resistance, and aerobic plus resistance), session frequency, session length, and imaging modality were not conducted due to insufficient data, so future studies are needed to clarify the effects of these factors.

## 5 Conclusion

This meta-analysis showed that exercise improves cardiac structure and systolic function parameters and VO_2_peak in T2DM patients, but does not significantly affect cardiac diastolic function parameters. HIIT seems to be superior to MICT for improving VO_2_peak and LVEF in T2DM patients. Future studies are anticipated to further elucidate the mechanisms underlying the effects of exercise.

## Data Availability

The original contributions presented in the study are included in the article/Supplementary Material, further inquiries can be directed to the corresponding author.

## Footnotes

1
https://www.Gradepro.org
