# Supplementary material for: Meta-analysis of the effects of different exercise modes on cardiac function and peak oxygen uptake in patients with type 2 diabetes mellitus
Source: Front Physiol. 2024 Nov 12;15:1448385. doi: 10.3389/fphys.2024.1448385 (PMC11588746; doi:10.3389/fphys.2024.1448385)

Additional file: fig.1 LVMI subgroup analyses


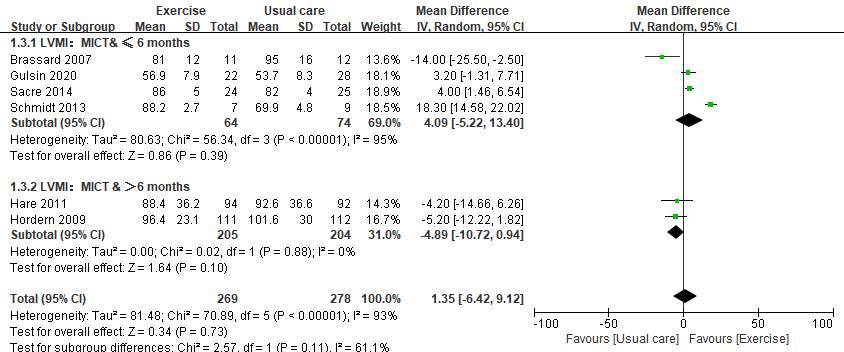


Additional file: fig.2 LVEF subgroup analyses


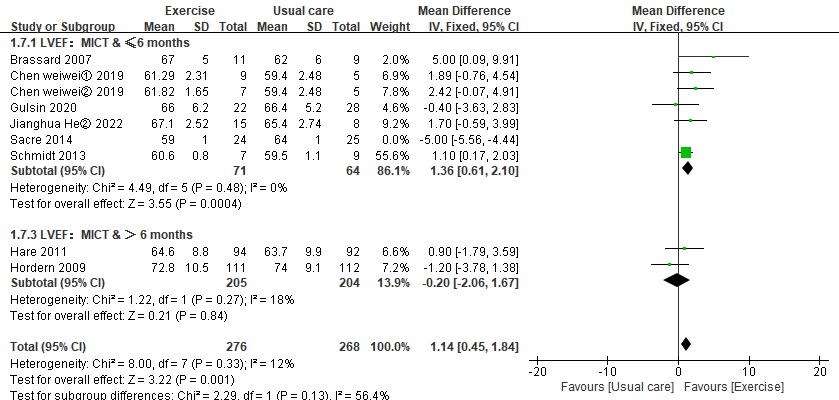


Additional file: fig.3 E subgroup analyses


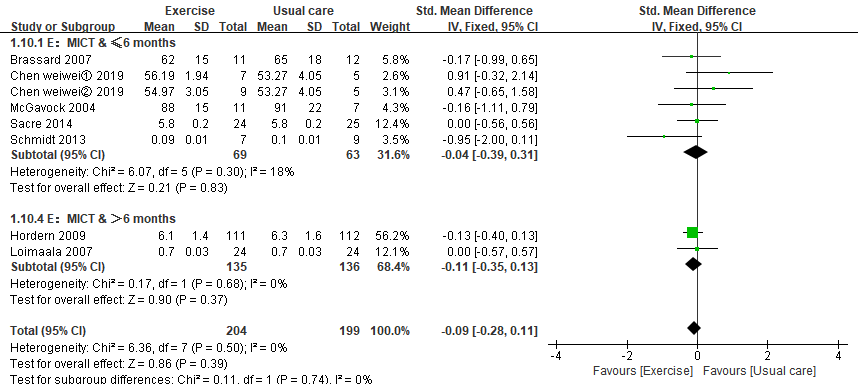


Additional file: fig.4 VO_2_peak subgroup analyses


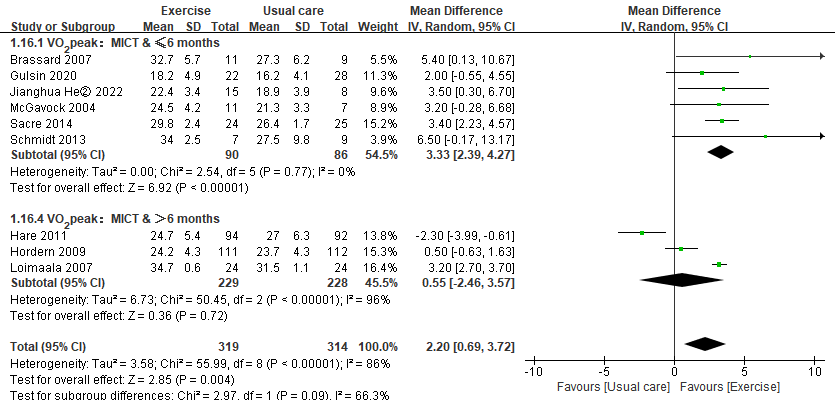


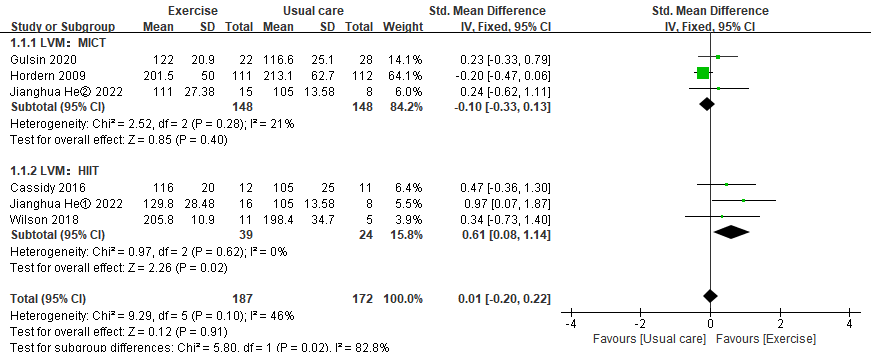


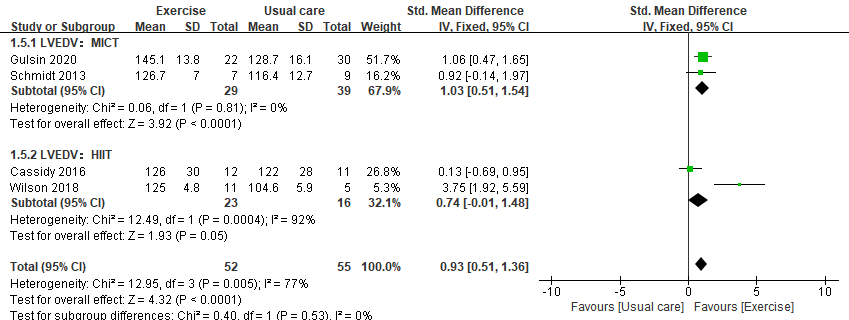


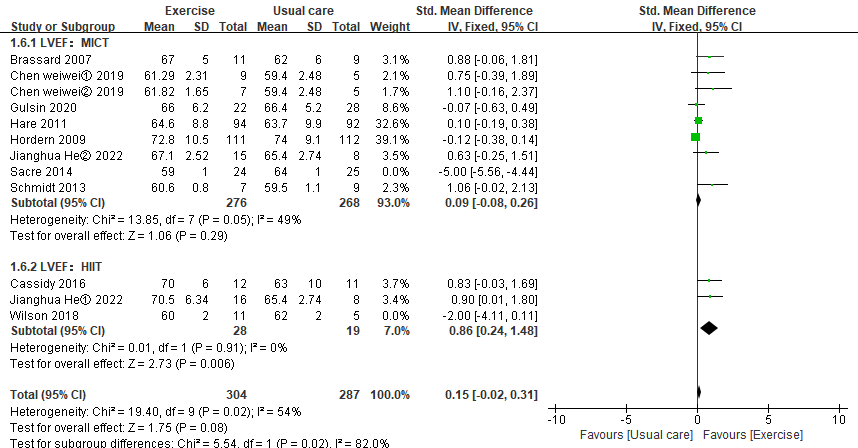


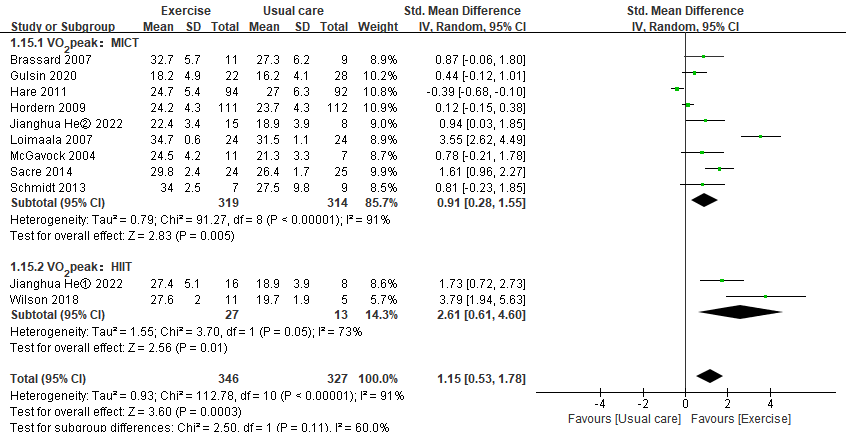


Additional file: fig.5 Sensitivity analysis

Additional file: fig.6 Risk of Bias


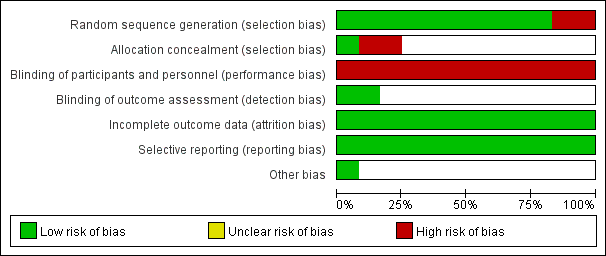


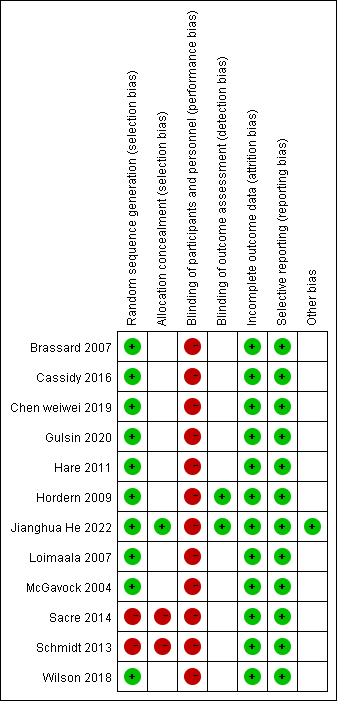


Additional file: fig.7 EBSCOhost search records


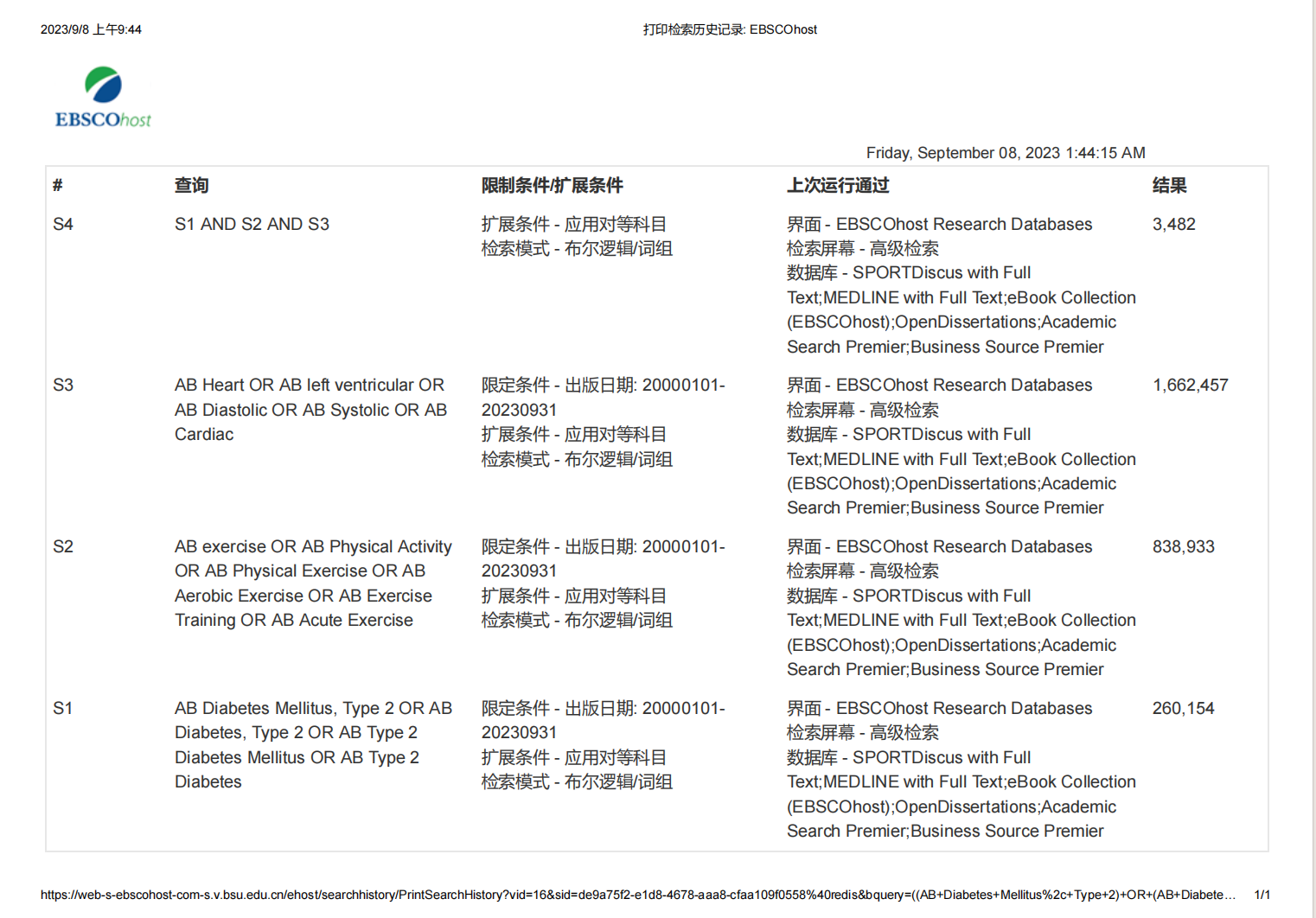

Supplement: Supplementary file 1 [file Table1.docx]
